# Proteomics Analyses of Small Extracellular Vesicles of Aqueous Humor: Identification and Validation of GAS6 and SPP1 as Glaucoma Markers

**DOI:** 10.3390/ijms25136995

**Published:** 2024-06-26

**Authors:** Raquel Rejas-González, Ana Montero-Calle, Alejandro Valverde, Natalia Pastora Salvador, María José Crespo Carballés, Emma Ausín-González, Juan Sánchez-Naves, Susana Campuzano, Rodrigo Barderas, Ana Guzman-Aranguez

**Affiliations:** 1 Chronic Disease Programme, UFIEC, Instituto de Salud Carlos III, 28220 Majadahonda, Madrid, Spain; raquel.rejas@isciii.es (R.R.-G.); ana.monteroc@isciii.es (A.M.-C.); 2 Biochemistry and Molecular Biology Department, Facultad de Óptica y Optometría, Universidad Complutense de Madrid, 28037 Madrid, Spain; 3 Analytical Chemistry Department, Facultad de CC. Químicas, Universidad Complutense de Madrid, Pza. de las Ciencias 2, 28040 Madrid, Spain; alejaval@ucm.es (A.V.); susanacr@quim.ucm.es (S.C.); 4 Opthalmology Service, Hospital Universitario Infanta Leonor, 28031 Madrid, Spain; npastora@ucm.es (N.P.S.); mcrespoc@salud.madrid.org (M.J.C.C.); emma.ausin@salud.madrid.org (E.A.-G.); 5 Oftalmedic Salvà, 07013 Palma, Illes Balears, Spain; juansanchez.naves@gmail.com; 6 CIBER of Frailty and Healthy Aging (CIBERFES), 28029 Madrid, Spain

**Keywords:** proteomics, mass spectrometry, glaucoma, cataracts, small extracellular vesicles, aqueous humor, glaucoma diagnosis

## Abstract

Cataracts and glaucoma account for a high percentage of vision loss and blindness worldwide. Small extracellular vesicles (sEVs) are released into different body fluids, including the eye’s aqueous humor. Information about their proteome content and characterization in ocular pathologies is not yet well established. In this study, aqueous humor sEVs from healthy individuals, cataracts, and glaucoma patients were studied, and their specific protein profiles were characterized. Moreover, the potential of identified proteins as diagnostic glaucoma biomarkers was evaluated. The protein content of sEVs from patients’ aqueous humor with cataracts and glaucoma compared to healthy individuals was analyzed by quantitative proteomics. Validation was performed by western blot (WB) and ELISA. A total of 828 peptides and 192 proteins were identified and quantified. After data analysis with the R program, 8 significantly dysregulated proteins from aqueous humor sEVs in cataracts and 16 in glaucoma showed an expression ratio ≥ 1.5. By WB and ELISA using directly aqueous humor samples, the dysregulation of 9 proteins was mostly confirmed. Importantly, GAS6 and SPP1 showed high diagnostic ability of glaucoma, which in combination allowed for discriminating glaucoma patients from control individuals with an area under the curve of 76.1% and a sensitivity of 65.6% and a specificity of 87.7%.

## 1. Introduction

Glaucoma and cataracts, which cause a progressive and severe loss of vision, are among the most remarkable eye pathologies. Glaucoma, the main cause of irreversible blindness, affects more than 70 million people, and its prevalence is estimated to increase to 112 million in 2040 [1]. Progressive death of retinal ganglion cells and their axons, accompanied by an ongoing loss of the visual field, characterize this pathology [2]. Elevated intraocular pressure (IOP) is considered an important risk factor for glaucoma; consequently, the therapies used are aimed at lowering IOP. However, the recurrence of patients who continue to lose vision even when IOP values are successfully controlled highlights the fact that elevated IOP is not the only determining factor in the development of the disease, and its control is not enough to delay or stop the course of the disease. IOP elevation can only be detected after significant visual field loss. In this sense, primary open-angle glaucoma (the most frequent) has been termed silent blindness since it usually does not produce symptoms until advanced stages. Therefore, there is a need to establish early diagnostic markers of glaucoma, as well as delve into the underlying causes of the disease. Cataracts, characterized by lens opacity, are responsible for 33% of visual impairment and 51% of blindness worldwide [3]. Although the main treatment for cataracts is surgery, there may be complications, such as a capsular tear of the lens or vitreous loss, as well as postoperative complications, such as endophthalmitis or posterior capsular opacification.

Considering the aging of the population worldwide, a higher prevalence of these eye diseases is expected in the future, which will imply a great economic cost for public health systems. In this context, diagnostic biomarker identification, analysis of pathological mechanisms and elucidation of potential therapeutic targets are a priority to improve the diagnosis and treatment of these ocular diseases.

Aqueous humor is a transparent fluid that fills the anterior eye chamber, is secreted by the ciliary epithelium, and drained by the trabecular meshwork and Schlemm’s channel. This ocular fluid provides nutrients to the trabecular meshwork, lens, and cornea, removes cellular waste products, and sustains intraocular pressure and refraction in the eye. It contains proteins and other molecules (lipids, dsDNA, ssDNA, miRNA…) released by intraocular structures from the anterior and the posterior segments of the eye. Due to its proximity to the pathology site and its enrichment with ocular tissue-specific proteins, aqueous humor represents an outstanding biological source for exploring pathological molecular mechanisms and biomarker identification in ocular diseases. Moreover, although it is usually collected during surgery, this fluid can be obtained safely and minimally invasively in patients through paracentesis [4]. It has been proposed that alterations in aqueous humor composition are associated with ocular pathologies development, and several reports have focused on mass spectrometry (MS)-based quantitative proteomics analysis of the aqueous humor protein profile in different ocular diseases. Thus, changes in aqueous humor proteome have been detected in patients with ocular pathologies such as cataracts [5,6], glaucoma [7,8], age-related macular degeneration [9], diabetic retinopathy [10,11], uveitis [12], and retinoblastoma [13]. In glaucoma studies, specific alterations in aqueous humor proteins have been demonstrated in patients with different types of glaucoma: primary open-angle glaucoma [14,15,16,17,18], primary angle-closure glaucoma [19], or pseudoexfoliation glaucoma [20]. Moreover, aqueous humor proteomic changes have been correlated with visual field indices, emphasizing their interest as diagnostic and prognostic biomarkers [21].

In the last few years, it has been established that some proteins and other molecules existing in the aqueous humor can be packaged into small extracellular vesicles (sEVs), whose presence in aqueous humor has been reported. sEVs are small lipid vesicles (<200 nm) that contain proteins, lipids, and nucleic acids [22]. Information about the release of sEVs by the different cell types present in the eye, their possible functions, or their involvement in ocular pathologies is still limited [23]. The vast majority of published studies focus on analyzing the types of existing extracellular vesicles (nanoparticle tracking) in aqueous humor [24,25], and in some cases, the miRNA content has been evaluated [26]. To date, only three articles have been published about the proteomic content of exosomes present in the aqueous humor, analyzing the protein content in sEVs derived from the aqueous humor of patients suffering age-related macular degeneration [27,28] and in patients with myopia [29]. On the contrary, there are no data about the sEVs protein content from glaucomatous aqueous humor, and information about the sEVs protein profile in aqueous humor from cataract patients is also scarce.

In this work, we have isolated and characterized aqueous humor sEVs from subjects with transparent lenses (ICL), cataract patients, and glaucoma patients. Moreover, an exhaustive characterization of the sEVs protein content from the aqueous humor has been performed using liquid chromatography-tandem mass spectrometry (LC-MS/MS). Significant changes in protein content were detected among the different groups of subjects, indicating sEVs protein profiles in these eye diseases. Validation of the results by WB and ELISA allowed us to identify growth arrest-specific protein 6 (GAS6) and osteopontin (SPP1) as relevant biomarkers for glaucoma diagnosis in aqueous humor with the high diagnostic ability of the disease.

## 2. Results

### 2.1. Workflow of Aqueous Humor sEVs Proteome Analysis

**Figure 1:**
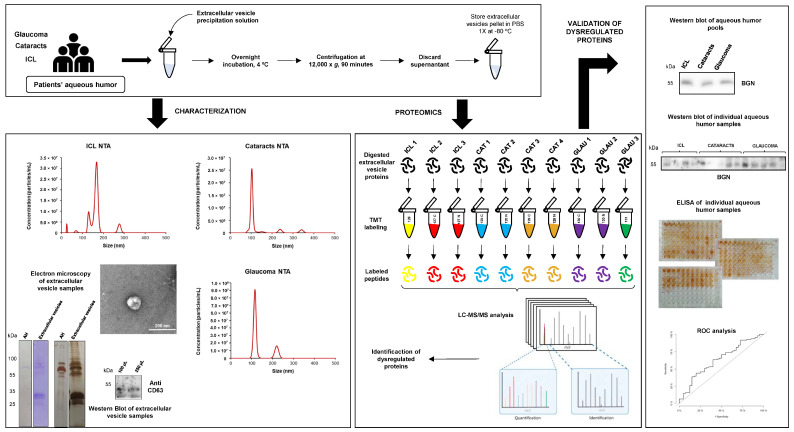
Workflow for the identification of dysregulated proteins from aqueous humor sEVs associated with cataracts and glaucoma. sEVs were isolated from pooled aqueous humor samples, and sEVs presence was analyzed by electron microscopy, nanosight technology (NTA), and western blot. sEVs proteins were digested with trypsin, labeled with TMT 10-plex, and analyzed by LC-MS/MS using an Orbitrap Exploris 480 coupled with the FAIMS Pro Duo Interface. After bioinformatics analysis of identified and quantified proteins, dysregulated proteins were selected for further validation by WB and ELISA. Finally, the diagnostic ability of selected markers was evaluated by ROC curve analyses using the concentration of the protein markers in the aqueous humor by ELISA. AH—aqueous humor; ICL—individuals with transparent lenses and without cataracts or glaucoma; CAT—cataract patients; GLAU—glaucoma patients. The average of three replicates of the same purified sEVs pool are represented in the NTA graphs.

In this work, we analyzed sEVs of aqueous humor as a barely analyzed source for the identification of glaucoma-associated dysregulated proteins that could be useful for its early detection. The sEVs used for proteomics analysis were isolated from three group conditions: cataracts, glaucoma, and ICL (individuals with transparent lenses and none of these pathologies). After TMT 10-plex mass spectrometry and bioinformatics analysis, dysregulated proteins were detected in the three groups with the MaxQuant software and the R program. Aqueous humor was used to validate the dysregulation observed at the sEVs level by WB analysis using individual or pooled samples. Finally, we analyzed the ability of GAS6 and SPP1 as glaucoma aqueous humor biomarkers to discriminate glaucoma patients from ICL and cataracts controls by ELISA using a collection of 162 individual aqueous humors samples (39 ICL, 92 cataracts, and 31 glaucoma samples) (Figure 1).

### 2.2. Characterization of sEVs Present in Aqueous Humors

For the identification of sEVs dysregulated proteins, aqueous humor samples from glaucoma patients (N = 13) and ICL (N = 19) were pooled into three pools, whereas samples from cataracts patients (N = 40) were pooled into four pools (Table 1 and Supplementary Table S1).

First, the extracellular vesicles present in the aqueous humor of glaucoma, cataracts, and ICL individuals were precipitated. Then, prior to further analysis by mass spectrometry, the quality of the isolated samples was analyzed to determine whether an appropriate isolation of the sEVs took place. To this end, and due to the scarce amount of sEVs samples obtained from aqueous humor pools, representative samples were analyzed by nanosight (NS300), electron microscopy (EM), and western blot (WB).

Isolated vesicles in suspension were observed using the NS300, obtaining the concentration and the mean size of the three samples analyzed and representative from the three groups of patients analyzed (Figure 1). The number of vesicles was more numerous in glaucoma patients (8.99 × 10^8^ particles/mL) than in cataracts and ICL control subjects (2.09 × 10^8^ and 5.12 × 10^8^ particles/mL, respectively). Moreover, the vesicles isolated from cataracts were more heterogeneous in size. However, the mean size of the vesicles in all samples ranged between 130 and 170 nm, which is in agreement with the size of the sEVs samples (Figure 1).

Next, by EM, a representative sample from cataracts allowed us to visualize vesicles no larger than 150 nm, compatible with the size of sEVs (Figure 1). Finally, we also examined the protein extract of these sEVs samples using Coomassie blue staining, silver staining, and WB. These analyses revealed differences in the protein content between the sEVs and the aqueous humor of the same patients’ pools. Notably, CD63 expression, a specific protein marker for extracellular vesicles, was observed in the sEVs samples. Collectively, these results confirmed an appropriate sEVs isolation from the aqueous humor.

### 2.3. Identification of Dysregulated Proteins from Aqueous Humor sEVs

After data normalization (Supplementary Figure S1), a total of 828 peptides and 192 proteins (Supplementary Table S2) were identified and quantified in the aqueous humor. Prior to the differential expression analyses, identified proteins were further investigated using different bioinformatic tools.

**Figure 2:**
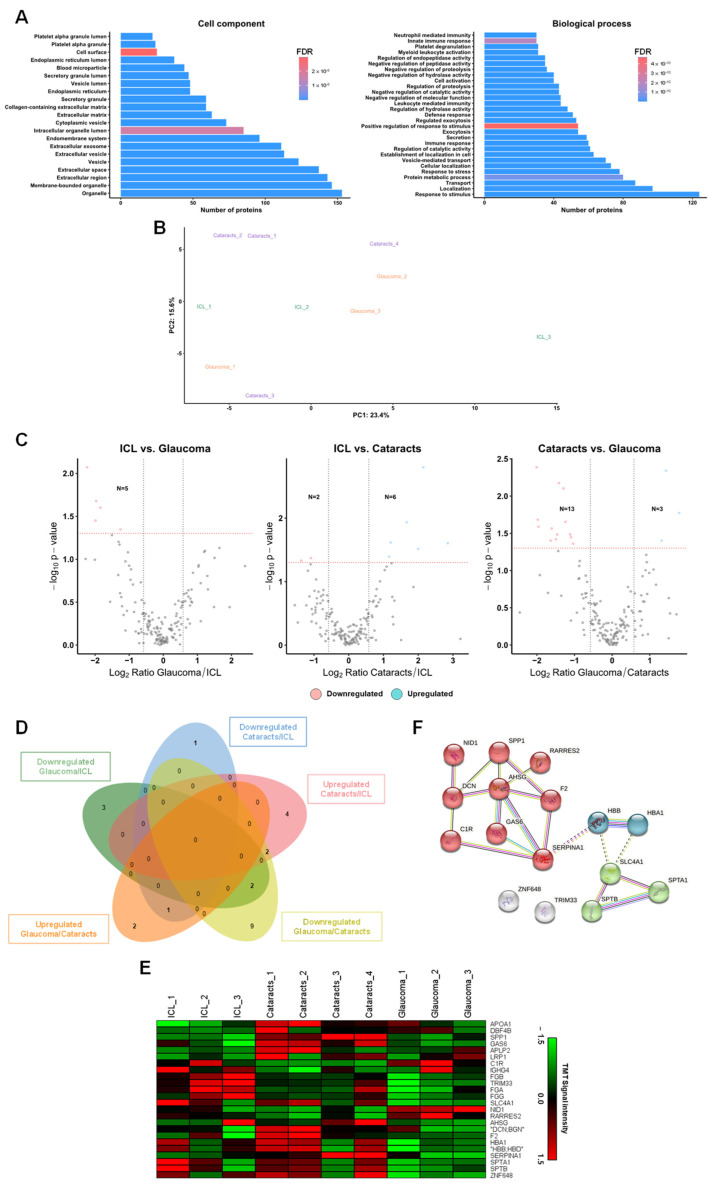
Analysis of the TMT 10-plex of aqueous humor sEVs. (**A**) Information on the subcellular location and the biological processes of the sEVs’ proteins identified in the proteomics analysis. (**B**) Principal component analysis (PCA) of the proteome of aqueous humor sEVs samples. (**C**) Volcano plots of the proteins identified as dysregulated in cataracts and glaucoma patients compared with ICL or comparing cataracts and glaucoma patients. (**D**,**E**) Venn diagram (**D**) and heat map (**E**) of the dysregulated unique proteins identified in all comparisons. (**F**) STRING revealed three clusters of interaction among the identified dysregulated proteins between cataracts and glaucoma. In red is the cluster associated with the response to stress and regulation of response to a stimulus. In blue is the cluster associated with cellular oxidant detoxification, nitric oxide transport, and carbon dioxide transport. In green, the cluster related to ankyrin binding.

First, gene ontology analysis was used for the identification of the enriched cell components and biological processes. Gene ontology revealed that more than 70% of identified proteins have been previously described as extracellular or exosome proteins (Figure 2A). Additionally, we also observed that most of the identified and quantified proteins were related to immune response, metabolic processes, or response to stimuli associated with the biological role of the aqueous humor (Figure 2A). These results confirmed the robustness of the identified and quantified proteins in the biological samples used, the appropriate isolation of sEVs from aqueous humor samples, and the quality of the sEVs used for LC-MS/MS analysis.

Next, by PCA analysis, we observed separated clusters for glaucoma, cataracts, and ICL control samples, suggesting the presence of differentially expressed proteins among groups (Figure 2B). Finally, although samples ICL_3 and Cataracts_3 grouped separated from their corresponding clusters, which might be a consequence of the interindividual heterogeneity of the patients included in each condition, all samples were used for data analysis and for the identification of dysregulated proteins in the three conditions. To this end, dysregulated proteins were identified using the moderated t-statistics expression ratios ≥1.5 (upregulated) or ≤0.67 (downregulated) and a *p*-value ≤ 0.05. In total, we identified five downregulated proteins in glaucoma sEVs and eight dysregulated proteins in cataracts sEVs (six upregulated and two downregulated) in comparison to sEVs from ICL individuals (Figure 2C). Interestingly, although only significant in the comparison of ICL vs. glaucoma, from the total number of dysregulated proteins, FGB was found downregulated in both pathologies when comparing to ICL samples (ICL vs. cataracts *p*-value 0.053, and ICL vs. glaucoma *p*-value 0.021), suggesting the potential of this protein as a biomarker of eye pathology. Furthermore, 16 proteins were found dysregulated (3 upregulated and 13 downregulated) in glaucoma sEVs in comparison to the sEVs from cataract patients (Figure 2C). These findings highlighted the potential of these proteins as aqueous humor biomarkers to discriminate cataracts or glaucoma patients from healthy ICL individuals (Figure 2D,E) as well as glaucoma from cataract patients.

Finally, since the most interesting comparison would be associated with the dysregulated proteins in glaucoma in comparison with cataract patients, the STRING database was used to examine clusters and networks where these 16 proteins would be involved. Interestingly, all proteins but ZNF648 and TRIM33 were grouped by direct and indirect interactions into three clusters. The main cluster is composed of nine proteins comprised dysregulated proteins related to response to stress and regulation of response to stimulus. The other two clusters, composed of two and three proteins, were mainly related to cellular oxidant detoxification and ankyrin binding, respectively (Figure 2F). These clusters, but ankyrin binding, would probably be associated with mechanisms of compensation to regulate the oxidative stress produced in glaucoma.

Collectively, we identified a total of 24 sEVs proteins by LC-MS/MS whose dysregulation was associated with cataracts and/or glaucoma diseases, and thus, with potential as biomarkers of these pathologies (Supplementary Table S3). Importantly, most of the proteins were previously found in sEVs of other pathological conditions, highlighting the robustness of the proteomics analysis performed.

### 2.4. Validation

Then, nine out of these proteins were selected for further validation of their role as cataracts and/or glaucoma biomarkers according to (i) their expression ratio in the three statistical analyses performed, (ii) their previous association with eye diseases, and (iii) antibody availability (Table 2). Moreover, as individual aqueous humor samples ranged between 25 and 150 µL, which prevented us from isolating sEVs from individual samples, we hypothesized that most of the dysregulated proteins in aqueous humor exosomes could be directly validated using aqueous humor samples and thus could serve as glaucoma biomarkers.

**Figure 3:**
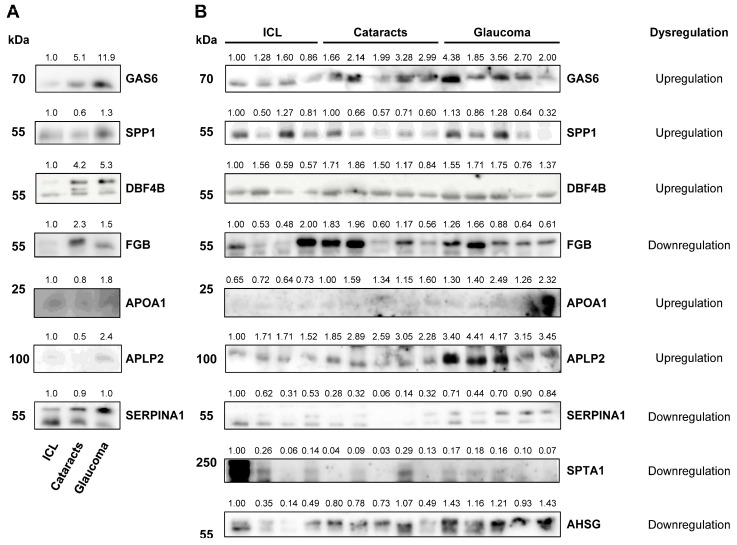
WB analysis of selected proteins in aqueous humor. (**A**) Pooled aqueous humors from ICL, cataracts, and glaucoma patients. (**B**) Individual aqueous humor samples from ICL (N = 4), cataracts (N = 5), and glaucoma (N = 5) patients. For each protein, the type of dysregulation detected in the TMT quantitative proteomic analysis is indicated. Coomassie blue staining and Ponceau red staining were used to compare and adjust band intensities.

To address this question, nine dysregulated candidate proteins (five upregulated and four downregulated) with available antibodies were selected for validation via WB analyses either using pools or individual samples from humor aqueous samples (Figure 3).

Despite not using sEVs but directly aqueous humor samples, WB results were certainly consistent with mass spectrometry quantification data. In this sense, the upregulation of APOA1 (apolipoprotein A-I) and APLP2 (amyloid-like protein 2) in cataract samples in comparison to ICL was confirmed in individual samples at similar extents to that obtained by mass spectrometry (Figure 3). In addition, upregulation of APOA1 and APLP2 in glaucoma samples was detected, as compared to ICL and cataracts. Moreover, the downregulation of SPTA1 (spectrin alpha, erythrocytic 1) and SERPINA1 (serpin family A member 1) was also confirmed in glaucoma patients in comparison to ICL and/or cataracts patients, as previously observed by mass spectrometry. Interestingly, WB analyses confirmed the dysregulation of GAS6 (growth arrest-specific 6), SPP1 (osteopontin), and DBF4B (DBF4 zinc finger B). Although some of these proteins exhibited a different trend than that expected in glaucoma pooled samples, WB results revealed the potential of these proteins as glaucoma biomarkers, underlying the importance of validation studies. On the other hand, FGB (fibrinogen beta chain) results showed a trend opposite to the one expected according to the proteomics results.

Collectively, although some differences were observed between proteomics data and WB, which would be related to the use of total aqueous humor fluid instead of their sEVs, immunostaining results allowed us to hypothesize that at least those upregulated proteins validated by WB in the aqueous humor could serve as glaucoma biomarkers.

### 2.5. GAS6 and SPP1 Analysis as Humor Aqueous Biomarkers for Glaucoma Diagnosis

To address this question, and as one of the goals of this study was to identify proteins dysregulated in aqueous humor that could be used as glaucoma biomarkers, we surveyed for differences in the protein levels of GAS6 and SPP1, using commercially available ELISAs in aqueous humor of glaucoma patients in comparison to cataracts and ICL patients. We focused on GAS6 and SPP1 because these validated dysregulated proteins in glaucoma by WB were among the most upregulated proteins found in aqueous humor.

**Figure 4:**
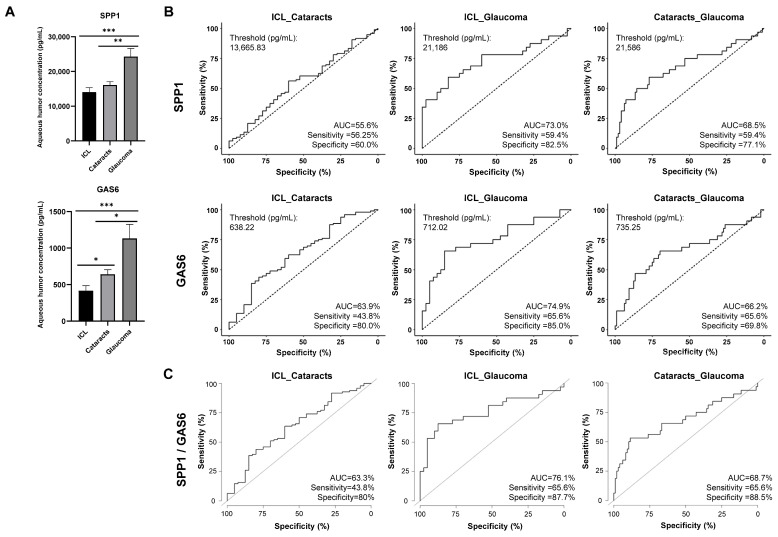
Analysis of SPP1 and GAS6 protein levels in aqueous humor samples from patients with cataracts, glaucoma, and ICL individuals. (**A**) Mean concentration of SPP1 and GAS6 in aqueous humor samples from ICL individuals (N = 39) and from cataracts (N = 96) and glaucoma (N = 31) patients, as determined by ELISA. (**B**) Individual ROC analyses of the diagnostic value of SPP1 and GAS6. (**C**) ROC analyses of the diagnostic value of SPP1 and GAS6 in combination. *, *p*-value < 0.05; **, *p*-value < 0.01; ***, *p*-value < 0.001; AUC—area under the curve.

Using 162 individual aqueous humors samples (39 ICL, 92 cataracts, and 31 glaucoma samples), the levels in aqueous humors of GAS6 and SPP1 significantly discriminated glaucoma patients from ICL or cataracts patients (control samples) (Figure 4). The mean concentration in pg/mL for GAS6 was 416.57 in ICL, 646.89 in cataracts, and 1185.36 in glaucoma samples. For SPP1, we obtained a mean concentration (pg/mL) of 14,063.73 in ICL samples, 16,080.71 in cataracts, and 24,279.04 in glaucoma patients.

Next, we determined the sensitivity and specificity of these candidate glaucoma biomarkers by ROC curves. Individual areas under the curve (AUCs) for discriminating glaucoma patients from ICL or cataracts were 73% and 68.5% for GAS6 and 74.9% and 66.2% for SPP1, respectively (Figure 4A,B). The combination of GAS6 and SPP1 increased the AUCs to 76.1% and 68.7% for discriminating glaucoma patients from ICL or from cataracts patients, respectively (Figure 4C), with a sensitivity of 65.6%, and a specificity of 87.7% and 88.5%, respectively. Moreover, we determined the cut-off concentrations (pg/mL) to differentiate glaucoma patients from ICL or cataract patients with ROC curves (Figure 4B).

These results confirmed the predictive value of GAS6 and SPP1 for the discrimination of glaucoma patients from ICL and cataracts individuals, which in combination improved their discrimination ability in terms of sensitivity and specificity. Our results highlight their usefulness in combination in the identification of early glaucoma patients using the aqueous humor after determining their concentration and classifying the individuals according to the determined cut-off value.

## 3. Discussion

There is an undeniable demand for glaucoma biomarkers to advance clinical testing for early diagnosis and disease progression follow-up.

Emerging evidence indicates that sEVs mediating intercellular communication could play a significant role in glaucoma. Thus, sEVs released by the ciliary body and the trabecular meshwork participate in the regulation of aqueous humor homeostasis [30]. In addition, extracellular vesicles from non-pigmented ciliary epithelial cells exposed to oxidative stress activate the antioxidant response of trabecular meshwork cells [31]. On the other hand, exosomes released by microglia challenged with high IOP increase the neuroinflammatory response in retinal cells and contribute to retinal degeneration [32]. These findings indicate that EVs and the molecular repertoire that they contain can represent a valuable source to deepen the knowledge of glaucoma pathology and identify new glaucoma biomarkers.

In this work, sEVs were isolated and characterized from aqueous humors of the different groups of study (ICL, cataract, and glaucoma patients). In agreement with previous reports [24], the number of sEVs, determined by nanoparticle tracking, was higher in glaucoma aqueous humor samples than in cataracts and ICL samples. Using mass spectrometry, the specific protein profile of sEVS from the ICL, cataract, and glaucoma samples was analyzed. Gene ontology analyses confirmed that more than 70% of the identified proteins corresponded to extracellular or EVs proteins, and, notably, it was observed, according to the ExoCarta database, that sEVs purified from aqueous humor contained many proteins previously described in sEVS from other cell types. Biological processes associated with most of the identified proteins were related to immune response, metabolic processes, and response to stimuli. Previous proteomics and gene ontology analyses of aqueous humor proteins related to cataract development revealed the association with metabolic processes [6], as well as immune responses and responses to stimuli [5]. Likewise, in addition to metabolic processes and stress response [15], a link between immune response activation and glaucoma aqueous humor proteins has been established in several studies [8,21].

Our differential expression analyses allowed us to determine five downregulated proteins in glaucoma sEVs and eight dysregulated proteins in cataracts sEVs as compared to sEVs from ICL individuals. Moreover, when both pathological groups were compared, 16 proteins were found dysregulated (3 upregulated and 13 downregulated) in glaucoma sEVs in comparison to the sEVs from cataract samples. Importantly, although data about aqueous humor sEVs protein profile in these pathologies are almost absent in databases, the dysregulated expression of some of these proteins has been previously determined in total aqueous humor from cataract and glaucoma patients. In this sense, IGHG4 (immunoglobulin heavy constant gamma 4) downregulation was detected here in sEVS from cataract’s aqueous humor as compared to ICL samples. Interestingly, this protein has been previously identified in total cataract aqueous humor and is considered a protein associated with cataract progression in three different studies [5,6,33]. Moreover, other previously identified cataract-related proteins, whose dysregulation was also observed in our study, were APLP2, FGB, APOA1, CRI (complement component 1R), and IGHG4 (immunoglobulin heavy constant gamma 4) [5,33].

Similarly to our findings, the upregulation of CRI (complement component 1R) and NID1 (nidogen 1) was detected in aqueous humor from several glaucoma types [17,34,35] as compared to cataract samples. Regarding CRI and NID1 upregulation, CRI is a protein of the complement system. There is growing evidence indicating that activation of the complement system contributes to retinal ganglion cell loss and glaucoma progression [36,37]. In the eye, nidogen 1 (NID1) is localized in trabecular meshwork [38] and seems to be critical for accurate retinal morphogenesis [39]. However, there is no information about its relationship with glaucoma.

Likewise, higher levels of SPP1 were found in aqueous humor from advanced normal tension patients [34]. In agreement with our results, the downregulation of FGA (fibrinogen alpha chain), FGG (fibrinogen gamma chain), and HBB (hemoglobin subunit beta) was assessed in primary open-angle glaucoma patients’ aqueous humor [8]. HBB protein levels seem to be positively correlated with visual field index parameters in glaucoma patients [21].

Additionally, although a different trend was reported, changes in the expression of F2 (prothrombin), AHSG (alpha 2-HS glycoprotein), and SERPINA1 have also been previously detected [17]. A factor that could have probably influenced this different trend is the distinct types of glaucoma considered in that study. Samples of acute angle-closure glaucoma, primary chronic angle-closure glaucoma, and neovascular glaucoma were evaluated, whereas primary open-angle glaucoma aqueous humor samples were used in our study.

Regarding the potential relation of these dysregulated proteins with glaucoma, changes in F2 could alter coagulation function, which is significantly associated with primary open-angle glaucoma patients and may contribute to primary open-angle glaucoma progression [40]. AHSG protein is a transforming growth factor (TGF)-β2’s antagonist [41]. Increased TGF-β2 expression reduces aqueous outflow by varying ECM composition in the trabecular meshwork, favoring IOP elevation and glaucoma [42,43]. Higher SERPINA1 concentration was assessed in the aqueous humor of patients with corneal allograft reversible rejection than that found in patients with irreversible rejection [44]. However, its exact relationship with glaucoma development remains unclear.

One of the goals of the study consisted of validating protein candidates in aqueous humor identified by mass spectrometry-based proteomics in sEVs. Thus, we tested the protein expression of selected sEV proteins in pooled and individual aqueous humor samples. All candidate proteins tested were detected in aqueous humor, showing a dysregulated profile. Immunostaining results were, in general, consistent with mass spectrometry quantification data, and the differences detected could be associated with the use of total aqueous humor instead of aqueous humor sEVs. In this study, although sEVs contain other biological macromolecules such as mRNA, miRNA, siRNA, or ssDNA that could also provide valuable information about the mechanism behind ocular diseases and the identification of biomarkers, we focused here on the analysis of sEVs protein content. Experimental work with sEVs from AH was challenging due to the small volume of AH per sEVs isolation. Consequently, this limits the approach to a more extensive analysis of the sEVs cargos. Future research is required to assess a broader range of sEVs molecular components.

The other main goal of the study consisted of trying to validate the dysregulated proteins as biomarkers of glaucoma in aqueous humor. To this end, we focused on GAS6 and SPP1, which exhibited a high upregulation in sEVs from aqueous humor from cataracts and/or glaucoma patients and were validated by WB in the total content of aqueous humor. Thus, the potential of these proteins identified in sEVs as glaucoma diagnostic biomarkers alone or combined was analyzed by ELISA. Remarkably, GAS6 and SPP1 were able to distinguish glaucoma patients from ICL and cataract patients with high diagnostic ability. Indeed, the combination of both biomarkers significantly improved the sensitivity and specificity to discriminate glaucoma patients from ICL and cataract patients, confirming the diagnostic usefulness of these aqueous humor proteins.

SPP1 is a member of the matricellular protein family that plays a fundamental role in extracellular matrix mineralization [45] and seems to show an interesting neuroprotective potential. SPP1 expression in α-retinal ganglion cells exposed to chronic IOP elevation promoted neuronal resiliency in glaucoma models [46]. In a similar way, SPP1 overexpression mediated by adeno-associated virus preserved retinal ganglion cells and vision in a mouse model of glaucoma induced by IOP elevation using microbeads injection [47]. Moreover, some studies have assessed SPP1 levels in primary open-angle glaucoma aqueous humor with opposite findings among them. Lower SPP1 levels were initially reported in glaucoma patients as compared to cataract patients [48], whereas, more recently, increased SPP1 concentration was found in glaucoma patients as compared to cataract patients [19,46].

In contrast, no information regarding the ability of GAS6 as a biomarker for glaucoma was found. GAS6 is a multimodular secreted protein that binds and activates the tyrosine kinase receptor family TAM (Tyro-3, Axl, and Mertk) [49]. In the retina, GAS6 has been identified as an angiogenic factor in zebrafish embryos and human retinal microvascular endothelial cells [50]. Increased expression of GAS6 has been described in the retina of an experimental model of multiple sclerosis, and its involvement in neuronal and glial cell survival has been determined in multiple sclerosis models [51]. Higher levels of GAS6 in cerebrospinal fluid of multiple sclerosis patients correlated with lower relapse severity [52]. Additionally, GAS6 is increased in cerebrospinal fluid of Alzheimer’s disease patients compared to controls, and, interestingly, patients with higher cerebrospinal fluid levels of this protein at diagnosis showed a less marked cognitive deterioration over a two-year follow-up [53]. Considering that glaucoma shares characteristics and pathological mechanisms with other neurodegenerative diseases [54], the upregulation of GAS6 expression found in glaucomatous aqueous humor could represent an attempt to moderate retinal ganglion cell damage counteracting glaucoma progression.

In summary, the elucidation of novel biomarkers with increased sensitivity and specificity remains a crucial challenge for early glaucoma diagnosis. This is a particularly important topic as the disease’s effects could be considerably lessened with early detection and appropriate management. In this work, we have isolated aqueous humor sEVs from ICL, cataract, and glaucoma patients and characterized their specific protein profiles. It allowed us to identify dysregulated proteins associated with glaucoma pathology that possess the diagnostic ability of the disease. Our findings revealed not only the interesting potential of sEVs aqueous humor for the determination of biomarkers with diagnostic interest but also the usefulness of GAS6 alone or in combination with SPP1 for glaucoma diagnosis.

## 4. Materials and Methods

### 4.1. Aqueous Humor Sample Collection

The clinical and ethical aspects of the study were approved by the Clinical Research Ethics Committee of Hospital Universitario Infanta Leonor (Madrid, Spain) (CEIC 011-23) and the Ophthalmedic and I.P.O. Institute (Palma de Mallorca, Spain). All patients provided written informed consent for the use of their biological samples for research purposes. The study adhered to the ethical principles promoted by Spain (LOPD 15/1999) and the European Union Fundamental Rights of the EU (2000/C364/01). All patient data were processed in accordance with the Declaration of Helsinki (last revised in 2013) and the Spanish National Biomedical Research Law (14/2007, of 3 July).

Aqueous humor was removed at the beginning of the surgery using lidocaine 2% (*w*/*v*) anesthetic drops and a 30-gauge Rycroft cannula attached to a 1 mL tuberculin syringe as previously described [55]. A volume of 0.05–0.15 mL of aqueous humor was collected per patient and stored immediately after surgery at −20 °C and subsequently at −80 °C.

The study comprised aqueous humor samples from subjects of 3 different groups: ICL (implantable collamer lens), cataract, and glaucoma (Supplementary Table S1). The ICL group included patients with a transparent lens (subjects without cataracts) who underwent refractive surgery involving the insertion of an intraocular lens. The cataract group included patients referred for cataract surgery who did not have glaucoma and had non-diabetic cataracts. The glaucoma group consisted of aqueous humor samples from glaucoma patients referred for cataract surgery. Pooled aqueous humor samples were used for the proteomics analysis, while both pooled and individual samples were used for validation (Supplementary Table S1 and Table 1).

### 4.2. Small EVs Isolation and Purification

For the aqueous humor samples, sEVs were isolated using the commercial precipitation solution ExoQuick (EXOQ20A-1, System Biosciences, Palo Alto, CA, USA). Samples of aqueous humor of the different conditions were pooled together according to their pathology state to a volume of 500 µL and then centrifuged at 4 °C, 3000× *g* for 15 min to remove cell debris. Subsequently, the precipitation solution was added to the supernatant and incubated overnight (O/N) according to the manufacturer’s specifications. Then, aqueous humors were centrifuged at 12,000× *g* for 90 min and 4 °C to precipitate the sEVs. After supernatant discard, pellets were resuspended in 250 µL of PBS 1X and stored at −80 °C until use. Finally, sEVs were diluted to 1:100 in PBS 1X for their analysis on a NanoSight NS300 (Malvern Panalytical, Malvern, UK) [56].

For the aqueous humor and sEVs samples, the protein concentration was assessed with the commercial MicroBCA Protein Assay Kit (Thermo Fisher Scientific, Waltham, MA, USA) and verified by Coomassie blue and silver staining [57,58].

### 4.3. Electron Microscopy

sEVs isolated from 900 µL of aqueous humor were resuspended in 110 µL of PBS 1X, and 10 µL was directly used for electron microscopy (EM) analysis with negative staining [56]. sEVs were fixed in PBS 1X and 4% of paraformaldehyde for 5 min and incubated on glow-discharged carbon-coated grids for 5 min. Then, samples were negatively stained using 2% aqueous uranyl acetate. Finally, samples were examined with an FEI Tecnai 12 electron microscope, and images were taken with an FEI Ceta digital camera [56].

### 4.4. Mass Spectrometry and Data Analysis

For the quantitative proteomics analysis, 5 µg of aqueous humor sEVs content per pooled sample was analyzed according to established protocols [56,58,59,60]. Aqueous humor sEVs were resuspended in 100 µL of RIPA prior to reduction with TCEP and alkylation with chloracetamide [60,61]. For a full description of the methodology, see [60,61].

The TMT experiment of aqueous humor sEVs was analyzed using an Orbitrap Exploris 480 mass spectrometer (Thermo Fisher Scientific) with FAIMS Pro Duo interface technology [61]. The Vanquish Neo UHPLC System (Thermo Fisher Scientific) was used for the peptide separation. For FAIMS, a gas flow of 4 L/min and CVs of −60 V and −45 V were used [61].

MS data analysis was performed with MaxQuant (version 2.1.3). Mass spectra files were searched against Uniprot UP000005640_9606.fasta Homo sapiens (human) 2022 database (20,577 entries, downloaded: March 2022) according to established protocols [60,61].

### 4.5. Normalization and Bioinformatics Analysis

TMT experiment normalization was made to equal the differences in the total sum of signals of each TMT channel, as the same amount of protein was labeled in each TMT sample. Sample loading (SL) and trimmed mean of M-values (TMM) data normalization was performed following established protocols with R Studio (version 4.1.1), using “tidyverse”, “psych”, “gridExtra”, “scales”, and “ggplot2” packages. To determine sample clustering, principal component analysis (PCA) was performed with the “stats” R package. Moderated t-statistics analysis was performed with R Studio packages “dplyr”, “tidyverse”, “limma”, “edgeR”, “ggplot2”, and “rstatix”. Proteins considered statistically significant dysregulated proteins were the ones identified with at least one unique peptide and an expression ratio ≥1.5 or ≤0.67 and *p*-value ≤ 0.05.

Dysregulated proteins were also analyzed using the STRING (version 12.0) to identify altered interacting clusters and networks related with these proteins. The STRING settings were established to Markov cluster algorithm (MCL) clustering enrichment 2 and a confidence score of 0.4. Protein enrichment was also studied with R Studio packages “STRINGdb” and “ggplot2”. The mass spectrometry proteomics data have been deposited at the ProteomeXchange Consortium via the PRIDE partner repository with the dataset identifier PXD048815.

### 4.6. SDS-PAGE and WB

For protein expression analysis, WB was performed after SDS-PAGE. A total of 5 µg of the aqueous humor samples (ICL N = 4, cataracts N = 5, and glaucoma N = 5) were loaded in a 10% SDS-PAGE and transferred to nitrocellulose membranes. For membrane blocking, 3% skimmed milk in PBS-Tween (0.1%) was used for 1 h. Subsequently, membranes were incubated with primary antibodies overnight at 4 °C (Supplementary Table S4). After three washes of 10 min with PBS-Tween 0.1%, incubation with the corresponding secondary antibody for 1 h was performed (Supplementary Table S4). After washes, an ECL Pico Plus chemiluminescent reagent (Thermo Fisher Scientific) was used to develop the signal, and an Amersham Imager 800 (GE Healthcare, Wauwatosa, WI, USA) was used to detect the signal and obtain the images. ImageJ software 1.54i was used for quantification of protein band intensities [58,62].

### 4.7. ELISA Tests

ELISA tests were performed to quantify the expression of GAS6 and SPP1 (DY885B and DY1433, R&D Systems, Minneapolis, MN, USA) in individual aqueous humor samples from ICL (N = 39), patients with cataracts (N = 92), and glaucoma patients (N = 31), according to established protocols [62,63,64]. In brief, 96-well microplates were coated with the capture antibody and incubated O/N at 4 °C. After washing, plates were blocked for 2 h with 200 µL of PBS 1X supplemented with 0.5% BSA at 37 °C. Washes were repeated after every incubation. Then, 2.5 µL of each aqueous humor sample diluted in PBS-BSA 0.5% were loaded per well and incubated for 1 h at 37 °C. Following another three washes, the detection antibody was added to the plates and incubated for 1 h. Then, plates were incubated with streptavidin-HRP at RT for 20 min at RT before the reveal. Protein concentration was evaluated by reading optical density at 495 nm in a microplate reader (Tecan Trading AG, Mannedorf, Switzerland).

### 4.8. Statistical Analyses

Microsoft Excel was used to obtain the mean, standard error of the mean (SEM), plots, and *t*-test. R Studio program (version 4.1.1) was used to obtain the values of non-parametric Mann-Whitney U tests and ROC curves (“ModelGood”, “Epi”, and “pROC” packages). *p*-values lower than 0.05 were considered statistically significant [56].

## 5. Conclusions

Glaucoma and cataracts are significant problems for public health. There is a clear need to delve into their pathogenesis and early diagnosis. Small extracellular vesicles (sEVs) present in aqueous humor could be a useful source for the identification of pathological molecular pathways and diagnostic biomarkers. Moreover, information regarding sEVs in the eye is scarce. We isolated and characterized aqueous humor sEVs from patients with glaucoma and cataracts and defined their specific sEVs molecular protein signatures using mass spectrometry. Furthermore, validation of the results directly with aqueous humor allowed us to identify GAS6 and SPP1 as relevant biomarkers for glaucoma diagnosis.

## 6. Patents

A patent in evaluation (P202430051) for glaucoma diagnosis based on the identification of GAS6 and SPP1 in aqueous humor resulted from the work reported in this manuscript.

## Figures and Tables

**Table 1:** Information on the aqueous humor samples used in the study.

Samples		Number (N)	Age Average ± SD (Years)	Min. Age (Years)	Max. Age (Years)	Gender (N)	
						M	F
TMT	ICL	19	42.1 (±11.42)	22	59	8	11
	Cataracts	40	67.27 (±10.03)	40	82	15	25
	Glaucoma	13	67.54 (±15.72)	26	89	4	9
WB	ICL	4	47.5 (±14.43)	26	57	1	3
	Cataracts	5	68.6 (±7.73)	60	79	3	2
	Glaucoma	5	66.4 (±12.66)	51	80	1	4
	Pool ICL	4	45.25 (±12.34)	31	57	1	3
	Pool cataracts	5	73.6 (±14.10)	55	89	2	3
	Pool glaucoma	4	62.25 (±11.00)	55	74	1	3
ELISA	ICL	39	43.38 (±12.05)	22	59	10	29
	Cataracts	96	69.32 (±10.67)	41	91	35	57
	Glaucoma	31	70.19 (±20)	26	89	10	21

**Table 2:** Information of the selected dysregulated proteins for validation identified by proteomics and bioinformatic analysis.

Protein Name	Analysis	Dysregulation	Log_2_ Ratio *	*p*-Value	Exocarta
APOA1	ICL–Cataracts	Upregulated	1.21	0.024	Colorectal cancer cells, liver cancer cells, melanoma cells, etc.
DBF4B	ICL–Cataracts	Upregulated	2.00	0.031	Mesenchymal stem cells
SPP1	ICL–Cataracts	Upregulated	2.15	0.002	Ovarian cancer cells
	Cataracts–Glaucoma	Downregulated	−1.42	0.007	
GAS6	ICL–Cataracts	Upregulated	1.17	0.049	Colorectal cancer cells, mesenchymal stem cells, nasopharyngeal carcinoma cells
	Cataracts–Glaucoma	Downregulated	−1.29	0.008	
APLP2	ICL–Cataracts	Upregulated	2.85	0.025	Colorectal cancer cells, ovarian cancer cells, saliva
FGB	ICL–Glaucoma	Downregulated	−1.97	0.021	Hepatocellular carcinoma cells, malignant pleural effusions, mesenchymal stem cells, plasma, etc.
SERPINA1	Cataracts–Glaucoma	Downregulated	−1.11	0.03	Colorectal cancer cells, hepatocellular carcinoma cells, liver cancer cells, plasma, platelets, urine, etc.
SPTA1	Cataracts–Glaucoma	Downregulated	−1.98	0.021	-
AHSG	Cataracts–Glaucoma	Downregulated	−1.96	0.026	B-cells, colorectal cancer cells, hepatocellular carcinoma cells, melanoma, mesenchymal stem cells, platelets, prostate cancer cells, urine, etc.

* Log_2_ Ratio of the indicated comparisons (i.e., cataracts–ICL, glaucoma–ICL, or glaucoma–cataracts).

## Data Availability

The data supporting the findings of this study are available from the corresponding author upon request. The mass spectrometry proteomics data have been deposited at the ProteomeXchange Consortium via the PRIDE partner repository with the dataset identifier PXD048815.

## Supplementary Materials

The supporting information can be downloaded at https://www.mdpi.com/article/10.3390/ijms25136995/s1.
